# An Improved Enzyme-Linked Focus Formation Assay Revealed Baloxavir Acid as a Potential Antiviral Therapeutic Against Hantavirus Infection

**DOI:** 10.3389/fphar.2019.01203

**Published:** 2019-10-16

**Authors:** Chuantao Ye, Dan Wang, He Liu, Hongwei Ma, Yangchao Dong, Min Yao, Yuan Wang, Hui Zhang, Liang Zhang, Linfeng Cheng, Zhikai Xu, Yingfeng Lei, Fanglin Zhang, Wei Ye

**Affiliations:** ^1^Department of Microbiology, School of Preclinical Medicine, Fourth Military Medical University, Xi’an, China; ^2^Department of Infectious Diseases, Tangdu Hospital, Fourth Military Medical University, Xi’an, China; ^3^School of Pharmaceutical Science, Shanxi Medical University, Taiyuan, China

**Keywords:** viral nucleic acid synthesis inhibitors, hantavirus, FFA, T-705, BXA

## Abstract

Hantaviruses, etiologic pathogens responsible for two severe human diseases, exist in areas ranging from Eurasia to America and remain global public health concerns. Conventionally, plaque formation assays have been used for hantavirus titering. However, hantaviruses replicate slowly within cells and produce minimal cytopathic effects, making this technique difficult to master. The improved enzyme-linked immunosorbent assay-based antigen detection method is easier to perform but is still time consuming. Here, we established an enzyme-linked focus formation assay (FFA) for Hantaan virus titering that is twice as fast as traditional assays. Moreover, using this method, we evaluated the effects of favipiravir (T-705) and another influenza virus drug, baloxavir acid (BXA), on hantavirus replication. We found that the endonuclease inhibitor BXA exerted similar anti-hantavirus effects as T-705. Overall, we developed a time-saving method for hantavirus titering and suggest BXA as a potential treatment choice for hantavirus-exposed individuals.

## Introduction

Hantaviruses (*Hantaviridae*, *orthohantavirus*) cause hemorrhagic fever with renal syndrome (HFRS) and hantavirus pulmonary syndrome (HPS) worldwide (Jiang et al., 2017). Accordingly, hantaviruses can be characterized as both Old World and New World viruses. The number of reported cases ranges from 100,000 to 200,000 annually, with mortality rates up to 15% for HFRS and 45% for HPS without treatment (Jonsson et al., 2010). Hantaan virus (HTNV), the prototypical hantavirus, causes more severe HFRS cases than other Old World hantaviruses. Annually, the reported HFRS cases in China account for more than 85% of cases worldwide. The genome of HTNV consists of three segments, the L, M, and S segments, which are named after their lengths (large, medium, and small, respectively); these segments encode viral RNA-dependent RNA polymerase (RdRp/LP), glycoprotein (GPC), and nucleocapsid protein (NP), respectively.

Currently, the absence of licensed therapeutics to treat severe hantavirus infection in humans underlines the need to develop new antiviral therapies. However, traditional methods, e.g., the plaque formation test (PFT), usually take approximately 10 days (Takenaka et al., 1985); thus, screening a potential compound with such methods is time consuming. The lack of cytopathic effects (CPEs) of hantaviruses in cultured cells also makes this method difficult to perform (McCaughey et al., 1999). Therefore, different methods have been developed to detect hantavirus replication, such as the improved PFT (Padua et al., 2015), the focus staining test (Tanishita et al., 1984), enzyme-linked immunosorbent assays (ELISAs) (Cheng et al., 2014), immunofluorescence assay (IFAs) (Yu et al., 2012), quantitative real-time PCR (qRT-PCR) (Jiang et al., 2013; Wei et al., 2013), flow cytometry (FCM) assays (Barriga et al., 2013), and in-cell western (ICW) assays (Ma et al., 2017). However, these methods have their own defects. For example, our laboratory utilized the most prevalent ELISA-based 50% cell culture infectious dose (CCID_50_) endpoint assay to detect the HTNV titer, and owing to the slow propagation of hantaviruses, this method took approximately 10 days to perform.

To improve measurement performance, we developed an improved focus formation assay (FFA) for HTNV titering that combines the benefits of PFT and immuno-based assays. The initial procedure is similar to that used in PFT. However, the procedure later simulates an IFA, which requires both cell fixation and target protein detection with a specific antibody. Moreover, unlike the ICW-based assay we previously established for HTNV (Ma et al., 2017), the FFA requires less antibody usage and yields the number of infectious virion particles excluding the calculated virus protein expression level.

In this study, we applied FFA-based HTNV titering for antiviral molecule evaluation and found that the broad-spectrum virus inhibitor T-705 showed a promising effect, similar to findings in previous studies. Furthermore, we also assessed the effect of baloxavir marboxil (BXM; formerly S-033188), a newly U. S. Food and Drug Administration (FDA)-approved influenza virus drug that is converted into the active form baloxavir acid (BXA; formerly S-033447) inside cells (Hayden et al., 2018), on HTNV. BXA, which was developed by Shionogi and Roche, targets the endonuclease domain of the PA subunit and inhibits the cap-snatching activity of influenza virus RdRp, thereby blocking the virus replication cycle (Noshi et al., 2018). In addition, BXA was recently approved by Japan and the FDA (Heo, 2018). Our results showed that BXA could also inhibit HTNV replication, suggesting potential therapeutic applications for BXA against HTNV or other hantavirus infections.

## Materials and Methods

### Cells, Viruses, and Drugs

Vero E6 cells [American Type Culture Collection (ATCC), CRL-1586] were stored in our laboratory and were cultured in Dulbecco’s modified Eagle’s medium (DMEM; HyClone) supplemented with 10% fetal bovine serum (Gibco) and 0.1% penicillin–streptomycin–gentamycin solution (Solarbio) at 37°C with 5% CO_2_. HTNV strain 76-118 was preserved in our laboratory and propagated in Vero E6 cells as previously indicated (Ye et al., 2015). T-705 (favipiravir, HY-14768, purity 98.89%) and BXA (HY-109025A, purity 99.71%) were purchased from MedChemExpress (NJ, USA). Carboxymethylcellulose sodium salt (CMC) was purchased from Sigma (21902). An aminoethyl carbazole (AEC) substrate set was obtained from BD Biosciences (551951).

### Antibody and Conjugation

A mouse monoclonal antibody (mAb) against hantavirus NP, 1A8, was produced in the laboratory and conjugated with HRP using a Lightning-Link^®^ enzyme horseradish peroxidase (HRP) labeling kit (Innova Biosciences, 701-0000) according to the manufacturer’s instructions. Briefly, the purified 1A8 antibody was added to the Lightning-Link^®^ mix and resuspended gently with a pipette. The mixture was left overnight at room temperature (RT). After conjugation, LL-Quencher reagent was added at a proportion of 1 µl for every 10 µl of antibody used. After 30 min, the mixture was supplemented with 50% glycerol and placed in a −20°C freezer for long-term storage.

### Western Blotting and IFA

Western blotting was performed according to a standard protocol. Briefly, Vero E6 cells were seeded in six-well plates and were mock infected or infected with 10-fold-diluted HTNV for 3 days. The cells were then washed with Dulbecco’s Phosphate-Buffered Sallines (DPBS) and lysed with Radio Immunoprecipitation Assay (RIPA) buffer. After bicinchoninic acid (BCA) quantification, 30 μg aliquots of lysate were boiled for 10 min, subjected to 12% SDS-PAGE, and transferred to Polyvinylidene fluoride (PVDF) membranes (Millipore, Billerica, MA, USA). The membranes were blocked with TBST containing 5% bovine serum albumin and then incubated with primary antibodies [anti-HTNV NP mouse mAb (1A8) and anti-beta actin mouse mAb (Proteintech)] followed by secondary antibodies conjugated with infrared dyes (Li-Cor Biosciences, Lincoln, NE, USA). The membranes were visualized using an Odyssey infrared imaging system (Li-Cor Biosciences).

For IFA, cells were seeded onto coverslips in 24-well plates at a confluence of 60–70%. After adherence, the cells were mock infected or infected with 10-fold-diluted HTNV for 2 h with rocking every 15 min. Three days post-infection (dpi), the cells were subjected to an IFA according to an established protocol (Ye et al., 2015). Briefly, the cells were fixed with 4% paraformaldehyde (PFA) for 15 min and then permeabilized with 0.5% Triton X-100. The cells were incubated with fluorescein isothiocyanate (FITC)-conjugated 1A8 mAb at 4°C overnight. Hoechst 33258 was used to stain the cell nuclei, and the samples were imaged using a BX60 fluorescence microscope (Olympus, Tokyo, Japan).

### FFA

Vero E6 cells were seeded onto 12-well or 24-well plates overnight and grown into confluent monolayers. Next, 500 µl (100 µl for 24-well plates) of 10-fold-diluted HTNV stock was added to each well and adsorbed at 37°C for 2 h with rocking every 15 min. Then, the virus was discarded, and each well was covered with 1.5 ml of 1.6% (w/v) CMC in maintenance culture overlay medium. At different times post-infection, the overlay was discarded, and the cell monolayer was fixed with 4% PFA for 20 min at RT. Following permeabilization with 0.5% Triton X-100 for 20 min, the plates were incubated with HRP-1A8 overnight at 4°C. After three washes, the plates were stained with a mixture of AEC chromogen (20 µl) and AEC substrate (1 ml) for 30 min at 37°C. Then, the plates were dried, and the foci were counted.

### Cell Counting Kit-8 (CCK8) Cell Viability Assay

Vero E6 cells were seeded in 96-well plates (1.2 × 10^4^ cells/well) and treated with different concentrations of T-705 and BXA. At 24, 48, and 72 h post-infection (hpi; only the 72 hpi data are shown), the medium was removed, 100 μl of DMEM and 10 μl of CCK8 reagent were added to each well, and the cells were cultured for an additional 4 h in the dark. The plates were then shaken for 1 min, and the absorbance (A) was measured at 450 nm using a BioTek Synergy HT microplate reader. Cell viability was calculated using the following formula: cell viability = [(As − Ab)/(Ac − Ab)] × 100%, where As denotes the absorbance of the experimental wells containing cells, medium, CCK8 solution, and drug; Ac denotes the absorbance of the control wells containing the same components as the experimental wells except for the drug; and Ab denotes the absorbance of the blank wells containing only medium and CCK8 solution.

### FFA-Based Antiviral Efficacy Evaluation

Vero E6 cells were seeded onto 12-well plates overnight and grown into confluent monolayers. Next, 500 µl of diluted HTNV stock (corresponding focus number: 10–50) was added and adsorbed at 37°C for 2 h with rocking every 15 min. Then, the virus was discarded, and each well was covered with 1.5 ml of 1.6% (w/v) CMC in maintenance culture overlay medium with the indicated concentrations of T-705 or BXA. At 5 dpi, the overlay was discarded, and the cell monolayers were treated as indicated in section 2.4.

### Structural Modeling

To explore the binding patterns of the active sites of endonuclease from influenza B virus (IBV) [Protein Data Bank (PDB): 6FS8], HNTV (PDB: 5IZE), and Andes virus (ANDV; PDB: 5HSB) with BXA, molecular docking analysis was performed with AutoDock software. The docking analysis revealed that the endonucleases from IBV, HNTV, and ANDV had similar binding patterns with BXA.

### Statistical Analysis

All data are expressed as the mean ± SD. Statistical analyses were performed using GraphPad Prism 7 (GraphPad Software, La Jolla, CA, USA). P < 0.05 was considered to indicate significance.

## Results

### Establishment of an Enzyme-Linked FFA for Detection of HTNV Replication

**Figure 1:**
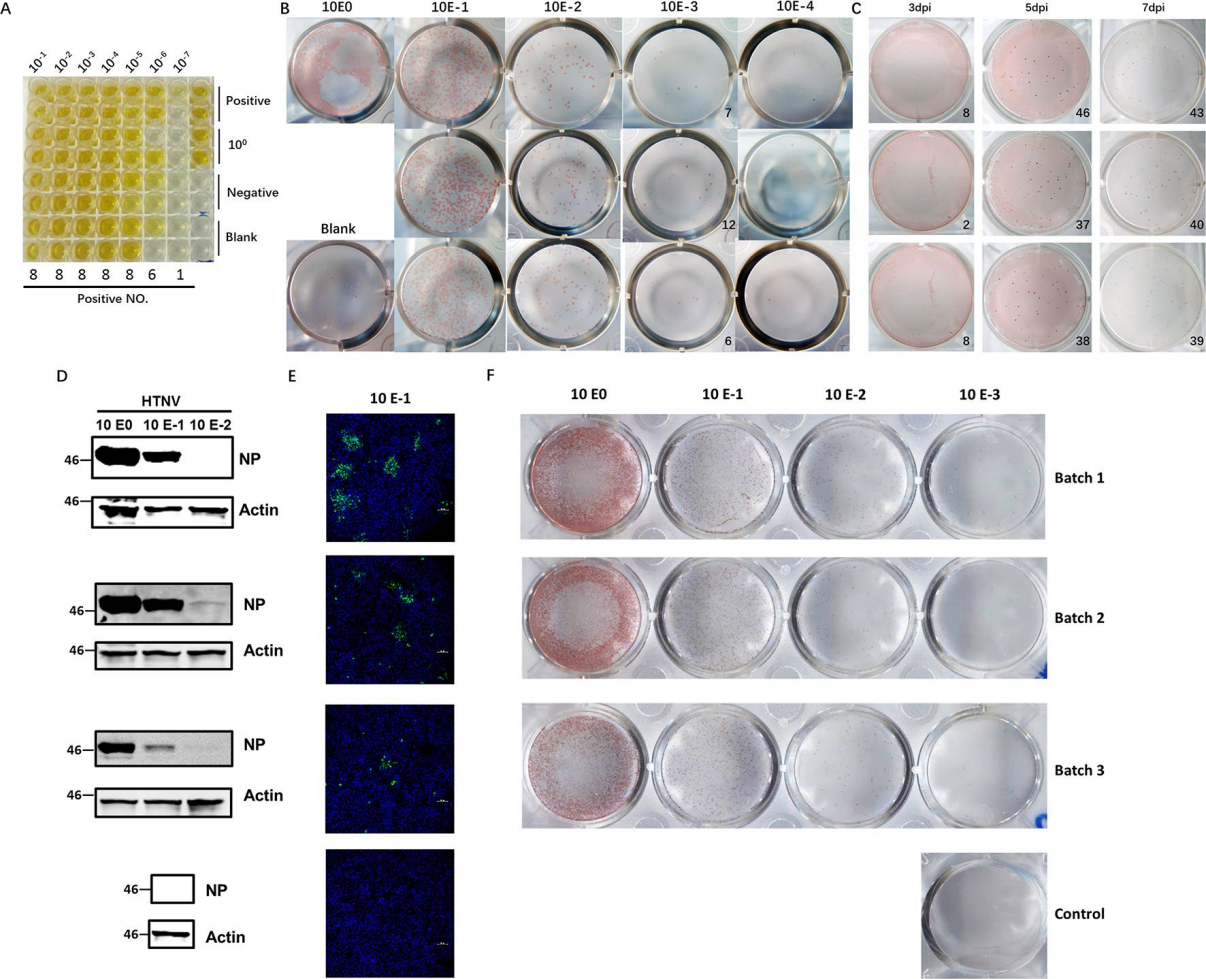
Application of the FFA to detect HTNV titers and its performance compared with that of conventional methods. **(A)** HTNV CCID_50_ titering by an ELISA-based method. Vero E6 cells in 96-well plates were inoculated with HTNV, maintained for 10 dpi, and subjected to three freeze/thaw cycles. Then, the supernatant was collected for titering. **(B)** Detection of the HTNV titer with the FFA method. Vero E6 cells in 12-well plates were inoculated with 10-fold-diluted HTNV and maintained with a CMC overlay. At 7 dpi, the FFA was performed with HRP-1A8 and AEC solution staining to assess HTNV NP expression. **(C)** Detection of the HTNV titer with the FFA method for a known CCID_50_ titer at different timepoints. Vero E6 cells in 12-well plates were inoculated with HTNV and maintained with a CMC overlay. At 3 dpi, 5 dpi, and 7 dpi, the FFA was performed. **(D)** Western blot measurement of NP expression with 10-fold-diluted HTNV at 3 dpi. Vero E6 cells in six-well plates were inoculated with 10-fold-diluted HTNV and maintained for 3 dpi, and 1A8 was used as the detection antibody. **(E)** IFA of NP expression with 10-fold-diluted HTNV at 3 dpi. Vero E6 cells on coverslips in 24-well plates were inoculated with 10-fold-diluted HTNV and maintained for 3 dpi, and 1A8 was used as the detection antibody. **(F)** FFA detection of the HTNV titer with 10-fold-diluted HTNV at 5 dpi. These experiments were performed independently at least three times with similar results.

Hantaviruses, known for their slow propagation and their failure to elicit observable CPEs, usually take at least 7–10 days to harvest, which makes them difficult to titer efficiently. The diagnostic method currently most often used involves detection of the most abundant antigen, NP, with a specific ELISA to measure the CCID_50_ titer and usually takes 10 days to perform. To establish a more accurate method for quantification of viable virus particles, we developed an FFA-based method for HTNV titering. First, we selected a virus stock with a known CCID_50_ titer (2.42×10^7^ CCID_50_/ml, **Figure 1A**). To use the FFA to evaluate the HTNV titer, we stained the plates at 7 dpi and counted the foci (**Figure 1B**). The FFA of this batch of virus stock was 8.3×10^4^ focus-forming units (FFU)/ml. The CCID_50_ titer and FFA titer were correlated; the titer obtained with the FFA was much lower than that obtained with the CCID_50_ method. Multiple experiments indicated that a range of 10–50 foci had the most consistent performance; thus, this range was used in subsequent tests.

To refine the optimal timepoints for FFA detection, we collected plates at different timepoints after virus inoculation. The foci were not visible in the 3 dpi group, but beginning at 5 dpi, the foci were sufficient for FFA detection (**Figure 1C**). There was no difference between 5 dpi and 7 dpi or later timepoints (data not shown); thus, the 5 dpi timepoint was used for subsequent detection, which cut in half the time required for the traditional ELISA-based method. To further evaluate the performance of the FFA-based HTNV titering assay, we compared the performance of different conventional methods with multiple batches of virus stock. As shown in **Figures 1D–F**, similar outcomes were obtained with the FFA method and the western blot and IFA methods. Therefore, this FFA method is suitable for rapid detection of slow-growing hantaviruses with high accuracy.

The homemade mAb 1A8 was generated using the traditional hybridoma method (Xu et al., 2002). Based on our previous data, 1A8 is applicable for detection of both HTNV and Seoul virus (SEOV) NP, the two most prevalent hantaviruses in China; thus, 1A8 mAb should be able to titer SEOV using the FFA. However, hantaviruses can be characterized into many different types worldwide. To test whether 1A8 mAb is suitable for detection of other hantaviruses, we tested the reactivity of 1A8 against the NPs of the most prevalent hantaviruses. As shown in **Figure S1**, 1A8 was suitable for IFA detection of HTNV, SEOV, and Dobrava-Belgrade virus (DOBV) but not Puumala virus (PUUV), Sin Nombre virus (SNV), ANDV, Prospect Hill virus (PHV), or Tula virus (TULV). Titering of these viruses may require other applicable NP antibodies.

### Application of the FFA for Evaluation of the Effects of Antiviral Molecules

**Figure 2:**
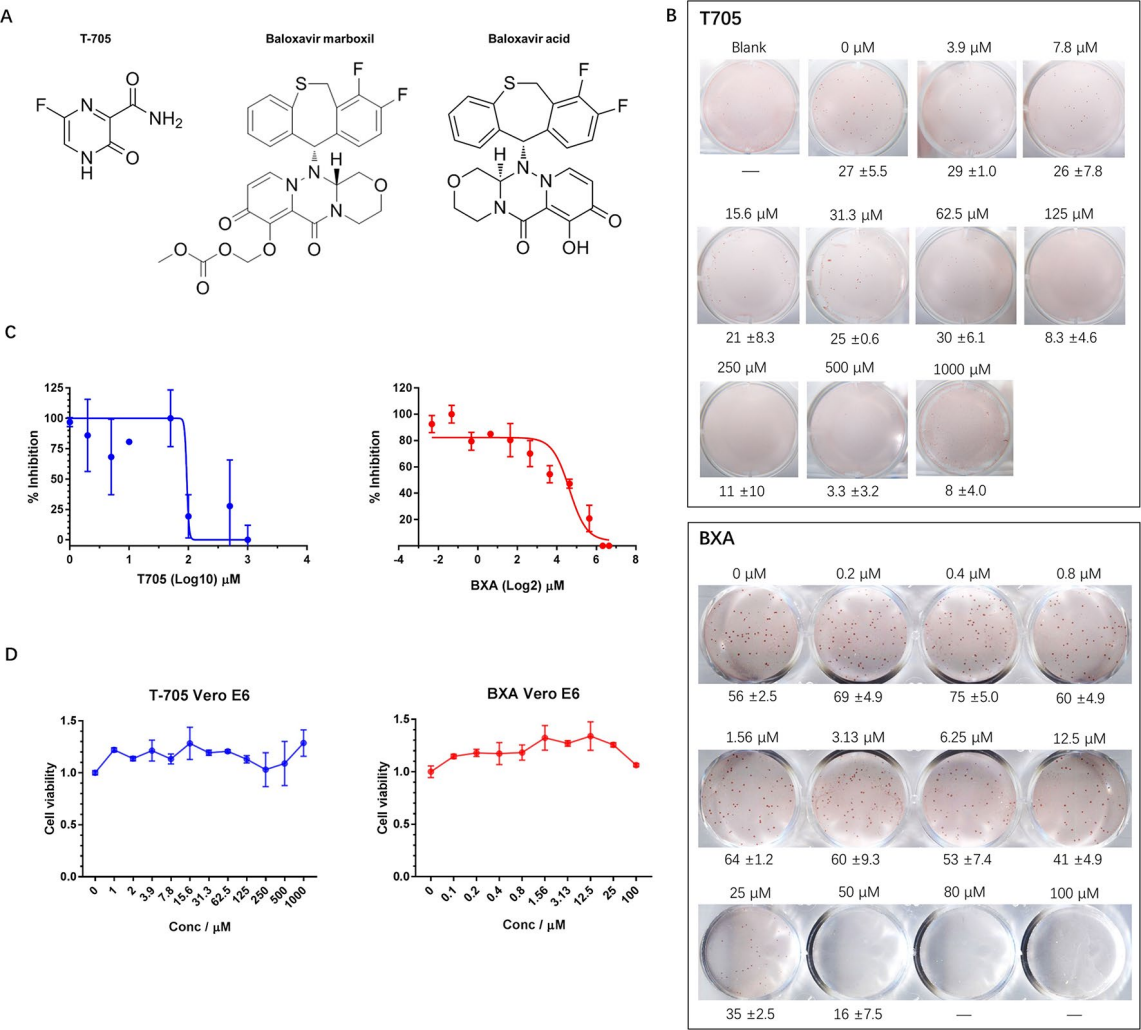
Application of the FFA to evaluate the effects of antiviral molecules on HTNV. **(A)** Chemical structures of T-705, BXM, and BXA. **(B)** Vero E6 cells in 12-well plates were infected with HTNV at an FFU of 30/well (T-705) or 50/well (BXA) and incubated with a CMC overlay supplemented with serial two-fold dilutions of inhibitors. At 5 dpi, the foci were counted and calculated. These experiments were performed independently at least three times with similar results. **(C)** Inhibitory effects of T-705 and BXA on HTNV replication, as measured by FFA. Vero E6 cells were infected with HTNV at an FFU of 30/well (T-705) or 50/well (BXA) and treated with serial two-fold dilutions of inhibitors. The foci were counted 5 dpi. Each point represents the mean and SD of three independent experiments. **(D)** Cell viability as a percentage of the control cell (treated with DMSO for T-705 or PBS for BXA) viability in uninfected Vero E6 cells incubated for 72 h post-T-705/BXA treatment. Each point represents the mean and SD of three independent experiments.

Currently, there are no approved therapeutics for hantavirus infection treatment; however, some compounds that possess potential anti-hantavirus effects have been identified. T-705, for example, is a nucleic acid mimic (**Figure 2A**) that was first developed for influenza virus infection treatment. Subsequent studies have shown that T-705 is capable of inhibiting RNA replication in multiple viruses regardless of its genome sense. Among these viruses, hantaviruses, including HTNV and ANDV, have been found to be inhibited by T-705 in the culture medium. We first determined whether the FFA method is suitable for hantavirus inhibitory compound screening and used T-705 as an indicator. When T-705 was added to the CMC overlay at different dilution rates, it showed an apparent concentration-dependent inhibitory effect on foci in wells infected with HTNV at the same multiplicity of infection (MOI). The 50% inhibitory concentration (IC_50_) of T-705 against HTNV determined by the FFA was 150.8 μM (**Figures 2B, C**). At this concentration, the drug showed no significant impact on cell viability (**Figure 2D**). Previous studies have also measured the IC_50_ of T-705 against hantaviruses using the focus formation method and have found that the IC_50_ for PHV is 66 μM, while that for DOBV is 93 μM (Buys et al., 2011). T-705 at a concentration of 25 μg/ml causes 100- to 1,000-fold reductions in both ANDV and SNV viral RNA levels in supernatants (Safronetz et al., 2013). These findings suggest that the FFA method is suitable for evaluation of the effects of antivirals.

BXM is an anti-influenza drug newly approved by the FDA for uncomplicated influenza treatment with only one dose (Hayden et al., 2018). BXM is transformed into its active form, BXA, by hydrolysis (**Figure 2A**) and targets the endonuclease domain of influenza virus RdRp. We hypothesized that, similar to T-705, BXA may inhibit hantavirus replication. To test this hypothesis, we evaluated the anti-hantavirus effects of BXA using the FFA method. BXA is capable of inhibiting influenza A virus (IAV), IBV, and some avian influenza viruses at a very low concentration; however, this was not the case for hantavirus. When BXA was added at a low concentration (≤1.56 μM) to the CMC overlay, the replication of HTNV in the treated group appeared similar to that in the control group. Only at high concentrations (≥6.25 μM) did we observe apparent inhibitory effects of BXA. The IC_50_ was 27.2 μM (**Figures 2B, C**), and it did not affect cell viability (**Figure 2D**). However, at very high concentrations (≥50.0 μM), prolonged incubation with BXA seemed to severely affect cell viability.

Thus, this newly established FFA method is suitable for screening potential therapeutics against hantaviruses. Using this method, we observed inhibitory effects of T-705 against HTNV replication and found that the newly approved influenza drug BXA is a possible anti-hantavirus compound.

### Possible Mechanism of HTNV Replication Inhibition by BXA

The mRNA generated by influenza virus RdRp is devoid of a 5’ cap, and the PA-PB1-PB2 heterotrimer is thought to be capable of digesting host mRNA and adding the host cap structure to its own mRNA for subsequent protein translation in a process called cap-snatching. Both hantaviruses and influenza viruses are segmented negative-sense RNA viruses, and this cap-snatching mechanism is thought to be conserved among negative-sense RNA viruses. Currently, there is no available structure for hantavirus RdRp. Under the new International Committee on Taxonomy of Viruses (ICTV) classification, the most closely related structure is that of another bunyavirus in the *Orthobunyavirus* genus, La Crosse virus (LACV). The structure of the large LACV RdRp is similar to that of the influenza virus PA-PB1-PB2 trimer and can also be characterized as having an RdRp domain at the C terminus and an endonuclease domain at the N terminus. The available structural information and functional experiment results regarding the N-terminal domain of hantavirus RdRp confirm the existence of an endonuclease domain.

**Figure 3:**
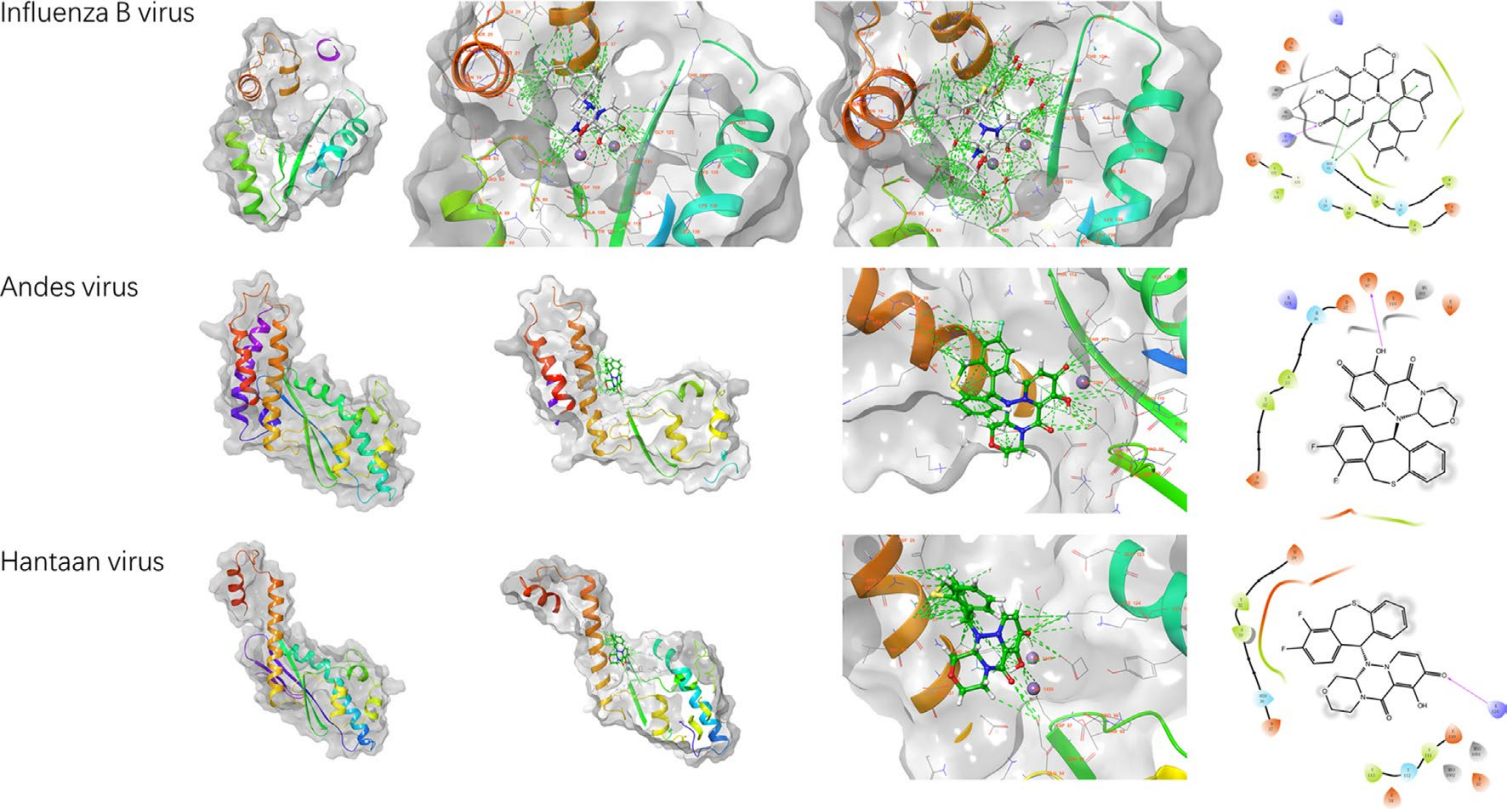
Structural modeling of the endonucleases from IBV (PDB: 6FS8), HNTV (PDB: 5IZE), and ANDV (PDB: 5HSB) with BXA using AutoDock software. The left three panels show the 3D structures. The left column shows the endonuclease domain of the RNA polymerase for each virus, the second column shows the molecule BXA modeled into the endonuclease domain, and the third column shows an enlarged view of the model, including the possible hydrogen bonds formed between BXA and the amino acids within the viral endonuclease domain. The right panels show the corresponding 2D interaction LIGPLOT schematics, which represent the possible interactions between viral amino acids and BXA.

To investigate the potential mechanism by which BXA inhibits hantavirus replication, the existing hantavirus endonuclease domain structure was used for structural modeling, and BXA was fitted into ANDV LP^endo^ and putative HTNV LP^endo^ structures similar to a structure obtained from IBV, as shown in **Figure 3**. Modeling provided only a preliminary mechanism for BXA inhibition of hantavirus replication; nonetheless, it is possible that BXA binds to the endonuclease domain of HTNV LP and exerts inhibitory effects. Taking this information into consideration for further improvement of the BXA compound may enable generation of more potent hantavirus inhibitors.

## Discussion

The high mortality and lack of effective approved treatments make hantavirus infection a public health threat worldwide (Jiang et al., 2017). Due to the slow propagation of hantaviruses and their failure to produce apparent CPEs, the current hantavirus titering methods usually take a week or more to perform. To enable discovery of new drugs that target hantaviruses, development of effective viral titering methods is a prerequisite. In this paper, we report a newly developed FFA-based approach to precisely titer HTNV. In addition, this method was used to evaluate the anti-hantavirus effects of two existing antiviral drugs.

The key concepts of this method are detection and visualization of HTNV NP. NP, the most abundant protein produced during hantavirus replication, serves as a marker for evaluation of virus replication levels and has been used in multiple different hantavirus titering methods. Compared to the traditional ELISA-based CCID_50_ method, the FFA method saves time and yields the precise number of infectious particles that exist in a virus stock. Thus, it is possible to measure non-CPE-producing viruses with accurate titers with this method. However, this FFA-based titering method also has its own defects; for example, CMC is quite viscous, and CMC overlay is relatively hard to master. In addition, the throughput is not high but can be upgraded using reagent-saving plates, such as 96-well plates. Compared to other methods, the FFA-based hantavirus titering method provides a more accurate way to evaluate viral titer.

To test whether this FFA method was suitable for antiviral molecule screening, we first evaluated the inhibitory effect of a known hantavirus inhibitor, T-705, on HTNV (Gowen et al., 2007; Buys et al., 2011; Safronetz et al., 2013). T-705 was first developed for influenza virus infection treatment and was subsequently found to have diverse virus-inhibiting functions (Furuta et al., 2013; Abdelnabi et al., 2017). The FFA results showed that T-705 can inhibit HTNV replication, consistent with results obtained with other methods (Gowen et al., 2007; Buys et al., 2011). Hence, the FFA-based method provides a time-saving and effective platform for antiviral drug screening.

A newly approved influenza drug, baloxavir, has a different mechanism than the neuraminidase-targeting oseltamivir (Heo, 2018). BXA derived from BXM is an endonuclease-targeting small molecule that inhibits influenza virus translation and thus inhibits virus replication (Heo, 2018; Omoto et al., 2018). Both hantaviruses and influenza viruses belong to the class of negative-sense RNA viruses, and the mRNA generated by the viral RdRp lacks a 5’ cap; therefore, these viruses must obtain a host mRNA cap and place it on their own mRNA (Reguera et al., 2010; Reich et al., 2014). BXA targets the endonuclease domain of the influenza virus PA-PB1-PB2 trimer. According to the existing structures of the RdRp molecules of another bunyavirus, LACV (Gerlach et al., 2015), and a mononegavirus, vesicular stomatitis virus (VSV) (Liang et al., 2015), among others, negative-sense virus RdRp molecules appear to share some key features. Within RdRp, the endonuclease domain seems more conserved than the other domains, especially within the order *Bunyavirales* (Amroun et al., 2017; Sun et al., 2018). Using an existing RNA endonuclease domain structure of ANDV LP and a modeled domain of HTNV (Fernandez-Garcia et al., 2016; Rothenberger et al., 2016), we fitted BXA into the active center of the endonuclease domain and found the fitness correlation with IBV. The modeling results may explain why BXA exerts anti-hantavirus replication effects. Moreover, this fitness may also extend to arenaviruses because of the structural similarity of the endonucleases between these viruses (Reguera et al., 2016).

Notably, the influenza epidemic suggests that BXA could select the direction of virus evolution and promote the I38T mutation (Takashita et al., 2019). A recent study already administered a combined treatment with BXA and a neuraminidase inhibitor to a mouse model (Fukao et al., 2019). However, such evolutionary pressure may not be a concern for BXA-based hantavirus therapeutics, as hantavirus infection occurs mostly through inhalation of rodent excreta, while person-to-person transmission is extremely rare; thus, the mutation rate could be less concerning for BXA-based hantavirus therapeutics than for BXA-based influenza virus therapeutics.

In brief, we applied an HRP-conjugated antibody to detect viral protein *in situ* and visualized the protein by staining it with the universally used Enzyme-linked Immunospot (ELISpot) substrate. Therefore, we established a novel, efficient, and time-saving FFA-based method to titer HTNV, a notoriously slow-growing and non-CPE-producing virus. Using this FFA method, we evaluated the anti-hantavirus eﬀects of two available molecules, T-705 and BXA. Both inhibitors efficiently inhibited HTNV replication; therefore, this approach provides a novel HTNV titering method that is also suitable for antiviral drug screening. In addition, the newly approved influenza drug BXA could serve as a lead compound with which to investigate new hantavirus-specific therapeutics.

## Data Availability Statement

All datasets generated for this study are included in the manuscript/**Supplementary Files**.

## Author Contributions

WY and DW performed the main experiments; CY executed the structural analysis; WY, YL, and FZ conceived and designed the research; WY, DW, and YL wrote the paper; WY, LZ, LC, ZX, YL, and FZ provided financial support; ZX, YL, and FZ provided administrative support; HL, HM, YD, LZ, MY, YW, and HZ participated in data collection and the provision of study material; and all authors read and approved the final manuscript.

## Funding

The present study was supported in part by grants from the National Key Research and Development Program of China (no. 2016YFC1202903), the National Natural Science Foundation of China (no. 31600131), the Key Research and Development Program General Project of Shaanxi Province (no. 2017SF-166), and the Open Fund of the State Key Laboratory of Pathogenic Microbial Biosafety (no. SKLPBS1834) and by a university supporting grant (no. 2018JSTS03). The funding bodies had no role in the design, interpretation, or submission of this work for publication.

## Conflict of Interest

The authors declare that the research was conducted in the absence of any commercial or financial relationships that could be construed as a potential conflict of interest.

## Supplementary Material

Figure S1 Detection of other hantaviruses using the mAb 1A8 with IFA A549 cells were seeded onto coverslips in 24-well plates at a confluence of 60–70%. After adherence, the cells were transfected with different hantavirus NP expression plasmids or vector controls. Twenty-four hours later, the coverslips were subjected to IFA as described above. The cells were incubated with the FITC-conjugated 1A8 mAb at 4°C overnight. Hoechst 33258 was used to stain cell nuclei, and the cells were imaged using a BX60 fluorescence microscope.
