# Corrigendum: An Improved Enzyme-Linked Focus Formation Assay Revealed Baloxavir Acid as a Potential Antiviral Therapeutic Against Hantavirus Infection

**DOI:** 10.3389/fphar.2020.00201

**Published:** 2020-02-27

**Authors:** Chuantao Ye, Dan Wang, He Liu, Hongwei Ma, Yangchao Dong, Min Yao, Yuan Wang, Hui Zhang, Liang Zhang, Linfeng Cheng, Zhikai Xu, Yingfeng Lei, Fanglin Zhang, Wei Ye

**Affiliations:** ^1^Department of Microbiology, School of Preclinical Medicine, Fourth Military Medical University, Xi'an, China; ^2^Department of Infectious Diseases, Tangdu Hospital, Fourth Military Medical University, Xi'an, China; ^3^School of Pharmaceutical Science, Shanxi Medical University, Taiyuan, China

**Keywords:** viral nucleic acid synthesis inhibitors, hantavirus, FFA, T-705, BXA

An author's name was incorrectly spelled as “Chuangtao Ye”. The correct spelling is “Chuantao Ye”.

The authors apologize for this error and state that this does not change the scientific conclusions of the article in any way. The original article has been updated.

