# Supplementary figures and images for: An Improved Enzyme-Linked Focus Formation Assay Revealed Baloxavir Acid as a Potential Antiviral Therapeutic Against Hantavirus Infection
Source: Front Pharmacol. 2019 Oct 16;10:1203. doi: 10.3389/fphar.2019.01203 (PMC6807675; doi:10.3389/fphar.2019.01203)

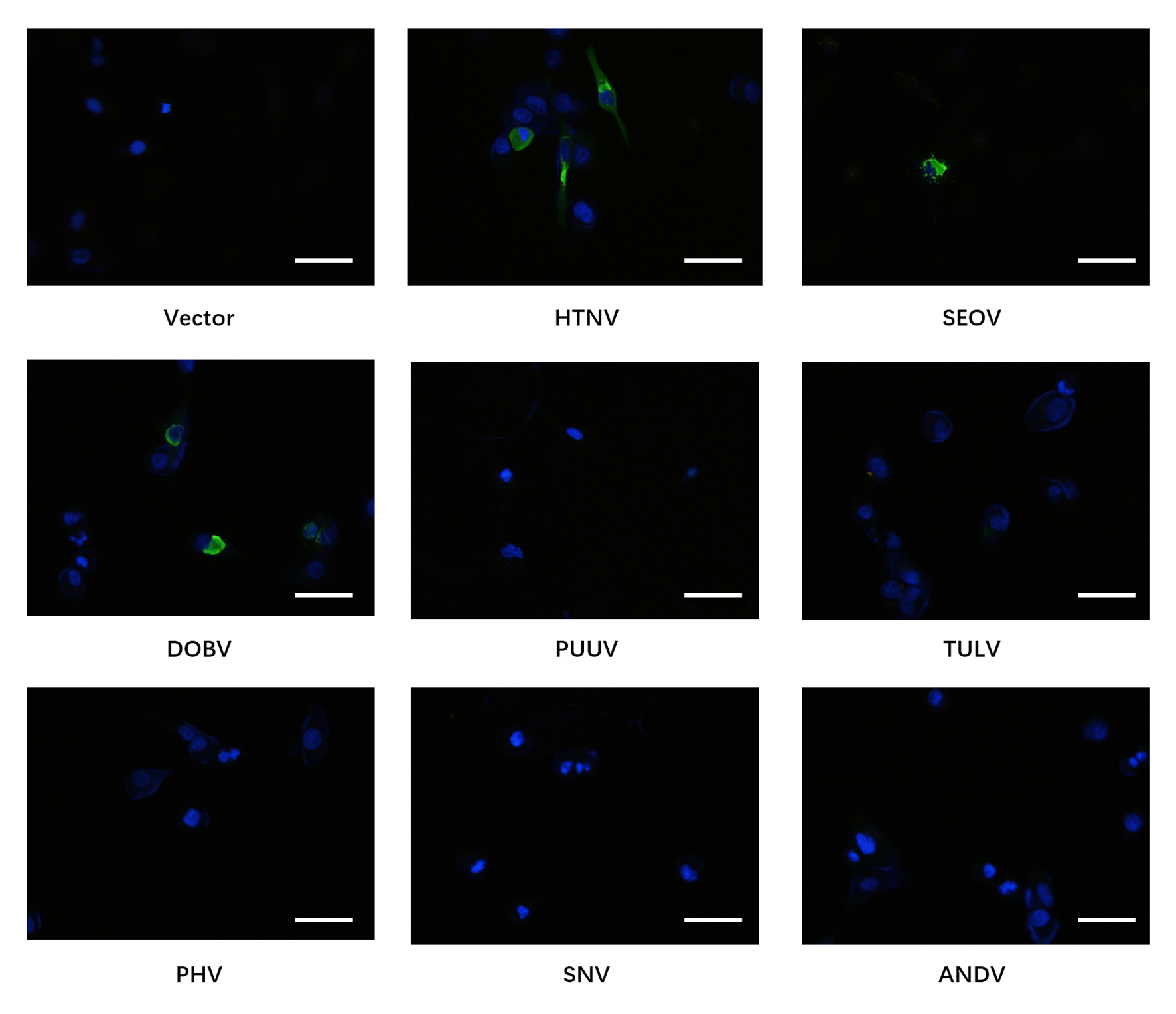

Supplement: Figure S1 — Detection of other hantaviruses using the mAb 1A8 with IFA A549 cells were seeded onto coverslips in 24-well plates at a confluence of 60–70%. After adherence, the cells were transfected with different hantavirus NP expression plasmids or vector controls. Twenty-four hours later, the coverslips were subjected to IFA as described above. The cells were incubated with the FITC-conjugated 1A8 mAb at 4°C overnight. Hoechst 33258 was used to stain cell nuclei, and the cells were imaged using a BX60 fluorescence microscope. [file Image_1.tif]
